# Combined inhibition of MEK and Aurora A kinase in *KRAS*/*PIK3CA* double-mutant colorectal cancer models

**DOI:** 10.3389/fphar.2015.00120

**Published:** 2015-06-16

**Authors:** S. Lindsey Davis, Kelli M. Robertson, Todd M. Pitts, John J. Tentler, Erica L. Bradshaw-Pierce, Peter J. Klauck, Stacey M. Bagby, Stephanie L. Hyatt, Heather M. Selby, Anna Spreafico, Jeffrey A. Ecsedy, John J. Arcaroli, Wells A. Messersmith, Aik Choon Tan, S. Gail Eckhardt

**Affiliations:** ^1^Division of Medical Oncology, Department of Internal Medicine, University of Colorado Anschutz Medical CampusAurora, CO, USA; ^2^University of Colorado Cancer Center, University of Colorado Anschutz Medical CampusAurora, CO, USA; ^3^Department of Pharmaceutical Sciences, Skaggs School of Pharmacy and Pharmaceutical Sciences, University of Colorado Anschutz Medical CampusAurora, CO, USA; ^4^Department of Drug Metabolism and Pharmacokinetics, Takeda California, Inc.San Diego, CA, USA; ^5^Department of Translational Medicine, Millenium Pharmaceuticals, Inc., A wholly owned Subsidiary of a Takeda Pharmaceutical Company LimitedCambridge, MA, USA

**Keywords:** MEK, Aurora A kinase, colorectal cancer, human tumor xenografts, alisertib, trametinib, KRAS mutation, PIK3CA

## Abstract

Aurora A kinase and MEK inhibitors induce different, and potentially complementary, effects on the cell cycle of malignant cells, suggesting a rational basis for utilizing these agents in combination. In this work, the combination of an Aurora A kinase and MEK inhibitor was evaluated in pre-clinical colorectal cancer models, with a focus on identifying a subpopulation in which it might be most effective. Increased synergistic activity of the drug combination was identified in colorectal cancer cell lines with concomitant *KRAS* and *PIK3CA* mutations. Anti-proliferative effects were observed upon treatment of these double-mutant cell lines with the drug combination, and tumor growth inhibition was observed in double-mutant human tumor xenografts, though effects were variable within this subset. Additional evaluation suggests that degree of G2/M delay and p53 mutation status affect apoptotic activity induced by combination therapy with an Aurora A kinase and MEK inhibitor in *KRAS* and *PIK3CA* mutant colorectal cancer. Overall, *in vitro* and *in vivo* testing was unable to identify a subset of colorectal cancer that was consistently responsive to the combination of a MEK and Aurora A kinase inhibitor.

## Introduction

With the advent of studies showing patients with *KRAS* exon 2 mutations (Amado et al., 2008; Karapetis et al., 2008), and now extended *RAS* mutations (*KRAS* exon 2, 3, 4, and *NRAS* exon 2, 3, 4) (Douillard et al., 2013; Heinemann et al., 2014), do not derive benefit from treatment with EFGR-targeting monoclonal antibodies, the treatment paradigm for metastatic colorectal cancer (CRC) is shifting to one focused on the molecular subsets of this malignancy. Not only do these studies demonstrate an unmet need for therapy in patients with extended *RAS* mutations, but also for those patients with *RAS* wild-type (WT) CRC who do not respond to EGFR-inhibition. The possibility of other molecular subtypes of CRC that may be represented within these unresponsive groups is of great interest, especially in the development of novel therapies for this disease. Given the lessons learned from the development of EGFR inhibitors in metastatic CRC, there is a new focus on identification of biomarkers predictive of response to novel agents early in their development.

Aurora kinase inhibitors are a class of novel agents that disrupt the normal functions of nuclear kinases Aurora A, B, and C in spindle pole organization and mitosis leading to disruption of cell division and chromosomal abnormalities (Lens et al., 2010; Kollareddy et al., 2012). Aurora A kinase-selective inhibitors are specifically known to induce transient mitotic arrest, with the goal of inducing apoptotic cell death in mitosis (Hilton and Shapiro, 2014). Clinical trials with these agents are still in early phases, though no overwhelming single-agent activity in colorectal cancer has yet been noted, and no biomarkers predictive of response to therapy have been identified (Diamond et al., 2011; Cervantes et al., 2012; Dees et al., 2012; Falchook et al., 2014). However, the specific, targeted mechanism of Aurora A kinase inhibitors makes their use in combination with an agent that may enhance apoptotic activity in cancer cells that have undergone abnormal mitotic progression one of great interest.

As *KRAS* mutations occur in approximately 40% of colorectal cancers (de Roock et al., 2010), various drugs targeting downstream of *RAS* in the mitogen-activated protein kinase (MAPK) pathway have been evaluated as potential therapies. MEK inhibitors, which have achieved significant success in the treatment of *BRAF* mutant melanoma (Flaherty et al., 2012), have been less effective as single agents in early clinical trials of both unselected (Bennouna et al., 2011) and *KRAS* mutant colorectal cancer patients (Zimmer et al., 2014). Though the use of MEK inhibitors as single-agent therapy in colorectal cancer is not supported by these results, a recent phase II study of a MEK inhibitor combined with irinotecan in *KRAS* mutant CRC yielded interesting results worthy of further study (Hochster et al., 2015). This suggestion of combination activity in a subgroup of CRC, as well as the known importance of the MAPK pathway in colorectal cancer, supports evaluation of MEK inhibitors as part of rational combination therapy with other novel agents.

Though the primary function of MAPK pathway inhibition is to block signaling for cell growth and proliferation, interruption of this pathway is also thought to affect cell cycle progression at G1 (Pages et al., 1993). In addition, it has been suggested that inhibition of MEK as an effector of the MAPK pathway affects the function of the cellular DNA damage response (Wei et al., 2011). It was thus hypothesized that adding a MEK inhibitor to an Aurora A kinase inhibitor, which may induce DNA damage through induction of defects in mitotic progression (Perez de Castro et al., 2013) and differentially target the cell cycle at G2/M phase (Hirota et al., 2003), may protect the genomic instability induced by the Aurora A kinase inhibitor from cell cycle check points and DNA damage response, ultimately facilitating cancer cell death (Collins et al., 2012). This hypothesis has been further supported by the concept that sustained cell cycle arrest (facilitated in this case by the combination of Aurora A kinase and MEK inhibition) allows for more consistent initiation of apoptosis and ultimately cell death (Hilton and Shapiro, 2014). The importance of the MAPK pathway in CRC, as well as the limited single-agent activity of MEK inhibitors in this tumor type, provide an ideal setting for evaluation of this hypothesis.

A recent study of Aurora A kinase inhibitor MLN8054, MEK inhibitor trametinib, and BRAF inhibitor dabrafenib evaluated various combinations of these drugs in *BRAF* mutant melanoma cell lines (Caputo et al., 2014). Of particular interest in this study were the more pronounced anti-proliferative effects of MLN8054 and trametinib as compared to the combination of trametinib and dabrafenib, a drug combination recently shown to improve progression free survival in patients with *BRAF* mutant melanoma (Long et al., 2014). Though observed in a different tumor type, the anti-proliferative effects of the combination of an Aurora A kinase inhibitor and MEK inhibitor demonstrated in this study do support further investigation of this novel combination.

The goal of this study was to evaluate the efficacy of the combination of an Aurora A kinase inhibitor and MEK inhibitor in colorectal cancer models, with an early focus on identification of a molecular subgroup of CRC more likely to benefit from such therapy.

## Materials and methods

### Reagents

Investigational Aurora A kinase inhibitor alisertib and MEK inhibitor TAK-733 were provided by Millennium Pharmaceuticals, and MEK inhibitor trametinib was purchased from Selleck Chemicals. For *in vitro* work all agents were dissolved in 100% DMSO at a concentration of 10 mM. For *in vivo* work, alisertib was suspended in a half volume of 20% 2-hydroxypropyl-β-cyclodextrin in sterile water (w/v) and then diluted with a solution of 2% sodium bicarbonate in sterile water (w/v) to provide a final formulation in 10% 2-hydroxypropyl-β-cyclodextrin / 1% sodium bicarbonate, TAK-733 was suspended in 0.5% methylcellulose, and trametinib was suspended in 10% Cremophor EL/10% PEG 400 in water.

### Cell lines and culture

The human colorectal cancer cell lines HCT116, DLD-1, LS174T, LS180, HCT-15, SW948, T84, Mip101, were obtained from American Type Culture Collection and SNU1544 was obtained from the Korean Cell Line Bank, and identities were confirmed by DNA profiling at the University of Colorado Cancer Center DNA Sequencing and Analysis Core. Cells were cultured in Roswell Park Memorial Institute (RPMI) media, supplemented with 10% FBS (Invitrogen), 1% nonessential amino acids (Cellgro Mediatech), 1% penicillin/streptomycin, and 0.1% puromycin. Cells were maintained in an incubator at 37° in 5% CO_2_.

### Evaluation of cellular proliferation

Sulforhodamine B (SRB) assay was used to evaluate proliferation of cells treated with TAK-733 and alisertib as single agents and in combination. Cells plated in tissue-treated 96-well plates were exposed to concentrations of single-agent TAK-733 increasing from 0 to 0.25 μM and to single-agent alisertib increasing from 0 to 0.5 μM. At 72 h cells were fixed with trichloroacetic acid (TCA) and stained with 0.4% SRB (MP biomedicals), with staining intensity quantified at an absorbance wavelength of 525 nM (Biotek Synergy 2). Cell lines were also exposed to combination therapy with TAK-733 and alisertib at the following doses: 0, 0.0625, 0.125, and 0.25 μM of TAK-733 and 0, 0.03125, 0.125, and 0.5 μM of alisertib. Proliferation evaluation was performed at 72 h by SRB assay as described above. The combination was evaluated for synergy using the Chou and Talalay method (CalcuSyn), with a CI value >1 consistent with antagonism, a CI value equal to 1 indicating additivity, and a CI value <1 indicative of synergy.

### Immunoblotting

Immunoblotting was performed on HCT116, DLD-1, LS180, and LS174T cell lines. Cells were seeded in 6-well plates and then treated with no drug, 0.125 μM alisertib, 0.125 μM TAK-733, or the drug combination at time points ranging from 24 to 72 h. At completion of drug exposure, media was aspirated and cells washed with 1X PBS. Radioimmunoprecipitation assay (RIPA) lysis buffer with Halt protease/phosphatase inhibitor cocktail (Thermo) was added for cell lysis, and protein quantified with use of Thermo Pierce 660 nm Protein Assay Reagent. Protein was run on 4–12% gradient Bis-Tris NuPAGE gels (Life Technologies) and transferred to nitrocellulose using the i-Blot system (Life Technologies). Primary antibodies used for immunoblot analysis include ERK, pERK, AKT, pAKT, Cyclin B1, pCyclin B1, PARP, Cleaved PARP, p53, p73, p21, pAurora A, pHH3, β-actin and α-tubulin (Cell Signaling Technology). Incubation with goat anti-mouse and goat anti-rabbit secondary antibodies (LI-COR Biosciences) followed. Blots were developed using the Odyssey Infrared Imaging System (LI-COR Biosciences).

For immunoblot analysis of phosphorylated Aurora A kinase, pre-treatment of cells with nocodazole was used to induce G2/M phase arrest. Cells were treated with nocodazole 0.5 μg/mL for 18 h, followed by a 2-h exposure to media without drug or with alisertib at a dose of 0.03125, 0.0625, or 0.125 μM. Immunoblotting was performed as above.

### Cell cycle analysis

Cell suspension containing 250,000 cells per well of HCT116, DLD-1, LS174T and LS180 was plated in 6-well plates for overnight incubation, and then treated with no drug, 0.125 μM alisertib, 0.125 μM TAK-733, or the combination for 24 h. Cells were then washed with 1X PBS and resuspended in Krishan's stain and incubated for 24 h at 4°C prior to flow cytometry. They were next analyzed by flow cytometry for cell cycle status by the University of Colorado Cancer Center Flow Cytometry Core Facility using an FC500 flow cytometer.

### Caspase 3/7 assay

DLD-1 and LS174T cell suspensions were plated in volumes of 100 μL into tissue-treated 96-well plates for overnight incubation. Cells were then exposed to 0, 0.03125, 0.125, or 0.5 μM of alisertib and 0, 0.0625, 0.125, or 0.25 μM of TAK-733 for 24, 48, or 72 h. A volume of 100 μL of Caspase-Glo 3/7 reagent was then added for incubation at room temperature for 1 h. Luminescence was then measured on a plate reader (Biotek Synergy 2) with normalization to the control group.

### Cell line xenograft models

Female athymic nude (nu/nu) mice were purchased from the Harlan Labs at age 4–6 weeks. Mice were allowed to acclimate for a minimum 7 days, and then caged in groups of 5 and kept on a 12-h light/dark cycle. They were provided sterilized food and water *ad libitum*. At logarithmic phase of growth, colon cancer cell lines HCT116, HCT15 and COLO741 were harvested. Cells were resuspended in a 1:1 mixture of serum-free RPMI media and Matrigel (BD Biosciences), and 2.5 × 10^6^ cells in a volume of 100 μL were injected into the right and left flank. Tumor sizes were monitored three times per week by caliper measurements with the Study Director Program, and tumor volumes calculated using the following formula: volume = (length × width^2^) × 0.52. Xenograft mice from each cell line were randomized into 4 groups once tumors reached 150–300 mm^3^: vehicle control, alisertib 3 mg/kg daily, TAK-733 3 mg/kg daily, or alisertib 3 mg/kg daily and TAK-733 3 mg/kg daily in combination. All drugs were administered via oral gavage. At the end of treatment (22 days for HCT116, 17 days for HCT15, and 29 days for COLO741), mice were euthanized with CO2 followed by cervical dislocation and tumor samples were collected. Animal studies were performed in a facility accredited by the American Association for Accreditation of Laboratory Animal Care in accordance with the NIH guidelines for care and use of laboratory animals and approved by the University of Colorado Institutional Animal Care and Use Committee (Permit number 51413(06)1E) prior to initiation.

### *In vivo* combination modeling

Tumors were modeled individually, with average model fits created from the mean of each individual fit, as previously described (Koch et al., 2009; Bradshaw-Pierce et al., 2013). Based on this modeling, a mathematical assessment of synergistic, additive, or antagonistic response to combination therapy was assessed. The mathematical term ψ was identified for each model, where ψ > 1.3 demonstrates a synergistic effect, ψ between 0.7 and 1.3 is consistent with additive effect, ψ between 0.7 and 0 is less than additive, and ψ < 0 is antagonistic. Additional modeling details can be found in the Supplemental Data Sheet 1.

### Patient-derived tumor xenograft models

Tumor specimens were collected from consenting patients at the University of Colorado Hospital at the time of surgery. Tumor tissue remaining after histopathologic analysis was cut into 2–3 mm^3^ pieces and submerged in Matrigel. Female athymic nude mice were acquired and cared for as described above. After a minimum of 1 week, 12 gauge trocars were used to inject tumor sections subcutaneously into the bilateral flanks of each mouse. Tumor sizes were monitored three times per week by caliper measurements as described above. Mice were randomized into 9 groups once tumors reached 150–300 mm^3^. These groups were then treated with vehicle control, alisertib 10 mg/kg daily, alisertib 20 mg/kg daily, trametinib 0.5 mg/kg daily, trametinib 1.5 mg/kg daily, or combination therapy with alisertib 10 mg/kg and trametinib 0.5 mg/kg daily, alisertib 10 mg/kg and trametinib 1.5 mg/kg daily, alisertib 20 mg/kg and trametinib 0.5 mg/kg daily, or alisertib 20 mg/kg and trametinib 1.5 mg/kg daily, all administered via oral gavage. After 32 days of treatment, mice were euthanized with CO2 and tumor samples were collected. As above, animal studies were performed in a facility accredited by the American Association for Accreditation of Laboratory Animal Care in accordance with the NIH guidelines for care and use of laboratory animals and approved by the University of Colorado Institutional Animal Care and Use Committee (Permit number 51413(06)1E) prior to initiation.

### Statistical analyses

A nonparametric Kruskal-Walls test with a Dunns post-test was used to determine statistical significance between multiple groups. An unpaired *t*-test with two-tailed *p*-values and 95% confidence interval was used to determine statistical significance between two groups. Analyses were performed with Prism version 5.0, *p* < 0.05 was considered statistically significant.

## Results

### *In vitro* effects of combination therapy with Aurora A kinase and mek inhibitors

#### Effects of alisertib and TAK-733 alone and in combination on proliferation of colorectal cancer cell lines

**Figure 1:**
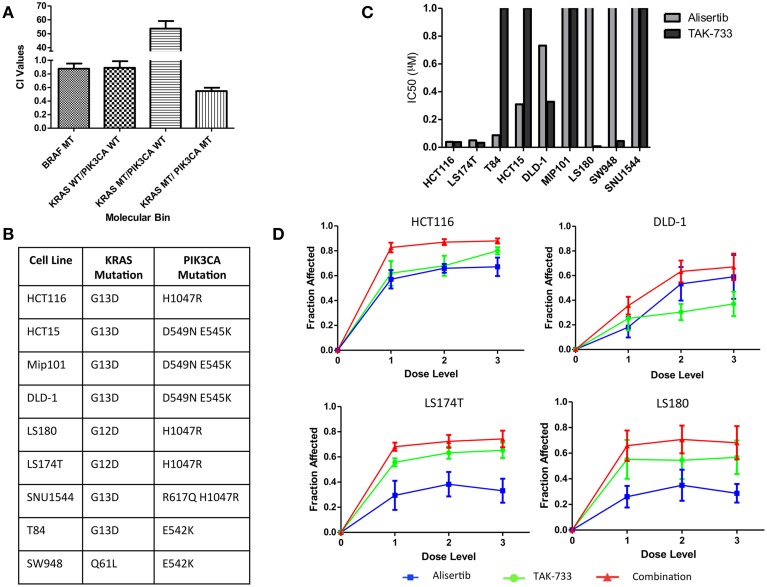
**Proliferative effects of alisertib and TAK-733 alone and in combination on colorectal cancer cell lines**. **(A)** Combination Index values for colorectal cancer cell lines of 4 distinct molecular subtypes treated with alisertib (0.05 μM, 0.1 μM, and 0.2 μM) and TAK-733 (0.06 μM, 0.125 μM, and 0.25 μM). Most pronounced synergy is observed in cell lines with *KRAS* and *PIK3CA* mutations, with a mean CI value of 0.55 ± 0.05, as compared to 0.876 ± 0.077 in the *BRAF* MT model, 0.89 ± 0.097 in the *KRAS* WT/*PIK3CA* WT model, and 53.68 ± 5.48 in the *KRAS* MT/*PIK3CA* WT model. **(B)** Mutation profile of 9 selected colorectal cancer cell lines with *KRAS* and *PIK3CA* mutations. **(C)** Calculated half maximal inhibitory concentration (IC_50_) values for *KRAS* and *PIK3CA* double-mutant cell lines treated with single-agent alisertib (doses ranging from 0.03125 to 0.5 μM) and TAK-733 (ranging from 0.06 to 0.25 μM). **(D)** Fraction of growth inhibition in double-mutant cell lines exposed to three different dose levels of alisertib and TAK-733 in combination by SRB at 72 h: 1 = alisertib 0.03125 μM and TAK-733 0.06 μM, 2 = alisertib 0.125 μM and TAK-733 0.125 μM, and 3 = alisertib 0.5 μM and TAK-733 0.25 μM.

Anti-proliferative effects of the combination of the investigational Aurora A kinase inhibitor alisertib and the MEK inhibitor TAK-733 were evaluated by SRB in cell lines representing 4 distinct molecular subtypes of CRC: *KRAS*/*PIK3CA* mutant (MT), *KRAS* MT/*PIK3CA* WT, *KRAS* WT/*PIK3CA* WT, and *BRAF* MT. Synergy was evaluated using the Chou and Talalay method (Chou and Talalay, 1984) in four cell lines from each molecular subtype; HCT116, HCT-15, Mip-101, and LS180 for *KRAS/PIK3CA* MT; GP5d, SW620, CL-11, and LoVo for *KRAS* MT/*PIK3CA* WT; HT55, HT15, SNU-1235, and SW48 for *KRAS* WT/*PIK3CA* WT; and COLO741, MDST8, HT-29, and RKO for *BRAF* MT. Average combination index (CI) values were compared between molecular subtypes. A CI value of <1 is consistent with a synergistic drug effect, while a CI value >1 is consistent with an antagonistic drug effect, and a CI value equal to 1 demonstrates an additive effect of the combination. A more pronounced synergistic effect was observed in the *KRAS*/*PIK3CA* MT (double-mutant) CRC cell lines, with a mean CI value of 0.55 ± 0.05, as compared to 53.68 ± 5.48 in the *KRAS* MT/*PIK3CA* WT model, 0.89 ± 0.097 in the *KRAS* WT/*PIK3CA* WT model, and 0.876 ± 0.077 in the *BRAF* MT model (Figure 1A). Based on these observations, the *KRAS*/*PIK3CA* MT molecular subtype was identified as one of particular interest in the evaluation of alisertib and TAK-733, and a total of nine double-mutant colorectal cancer cell lines were selected for further assessment (Figure 1B).

The nine additional CRC cell lines were exposed to each drug as a single agent, and proliferation was assessed to determine single-agent activity (Figure 1C). The HCT116, LS174T, and T84 cell lines were most sensitive to alisertib as a single agent, with calculated IC_50_ values of 0.04, 0.05, and 0.09 μM, respectively, while the LS180, LS174T, HCT116, and SW948 cell lines were most sensitive to single-agent TAK-733, with calculated IC_50_ values of 0.008, 0.033, 0.038, and 0.045 μM. When cells were exposed to the combination of alisertib and TAK-733 for 72 h, growth inhibition was noted to increase beyond that observed with either drug as a single-agent (data from 4 representative cell lines shown in Figure 1D). These effects were most consistently demonstrated at the middle (0.125 μM of alisertib and TAK-733) and high (alisertib 0.5 μM and TAK-733 0.25 μM) concentrations of each drug, and the middle dose level was selected for further investigation.

The effect of the drugs in combination was also evaluated through application of the Chou and Talalay method (Flanigan et al., 2013) to SRB proliferation data. Combination index values were compared between cell lines in an effort to identify subgroups within the double-mutant models in which synergistic vs. antagonistic effects of the drug combination might be identified. According to SRB data, a sub-additive response to combination therapy was demonstrated in the LS180, SW948 and SNU1544 cell lines, while synergy was demonstrated in all other cell lines (Supplemental Data Image 1). Four of the 9 double-mutant cell lines with varied proliferation data by SRB (HCT116, DLD-1, LS174T and LS180) were selected for further *in vitro* evaluation.

#### Effects on downstream effectors of MEK and Aurora A kinase inhibition in double-mutant colorectal cancer cell lines

**Figure 2:**
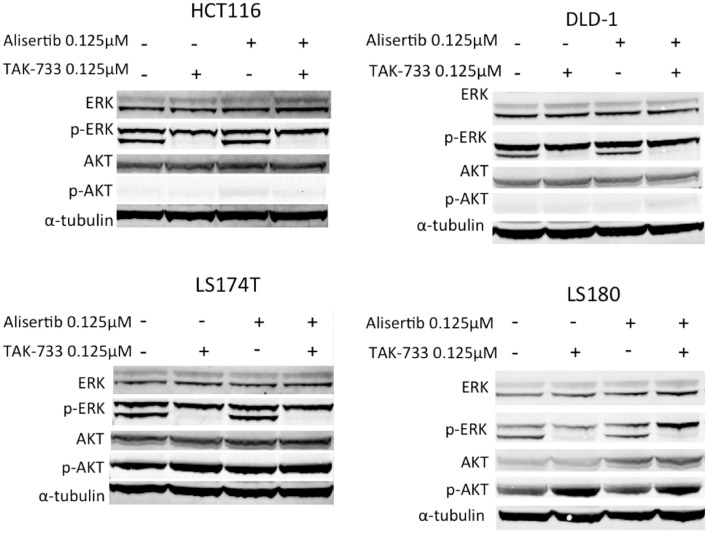
**Effect of MEK inhibition by TAK-733 alone and in combination with alisertib on downstream effectors**. Effectors of the mitogen activated protein kinase (MAPK) and phosphatidylinositide 3-kinase (PI3K) pathways were evaluated at 24 h. Phosphorylated ERK decreases in all double-mutant CRC cell lines when treated with TAK-733 alone or in combination with alisertib.

Immunoblotting for total and phosphorylated ERK was performed to evaluate MAPK pathway inhibition. As expected, cell lines treated with TAK-733 as a single agent demonstrated a clear decrease in phosphorylated ERK, which was maintained in the combination (Figure 2) (von Euw et al., 2012). Interestingly, in the LS180 cell line, an increase in phosphorylated AKT was observed following treatment with TAK-733 that was not observed in the other cell lines (Figure 2). Alisertib did not affect these MAPK pathway markers.

The effects of Aurora A kinase inhibition were also evaluated by immunoblotting, with use of nocodazole to facilitate arrest of cells in G2/M, the phase of the cell cycle during which Aurora A protein levels peak (Crosio et al., 2002). A decrease in phosphorylated Aurora A was observed in HCT116 and DLD1 cell lines, although this decrease was not as pronounced in the LS174T and LS180 cell lines (Supplemental Data Image 2). Levels of phosphorylated histone H3 (pHH3) were relatively stable in the treated HCT116, DLD-1, and LS174T cell lines. Of note, pHH3 did appear to decrease in LS180 when treated at 0.03125 and 0.0625 μM concentrations of alisertib as compared to nocodazole-only treated cells.

#### Effects of alisertib and TAK-733 on the cell cycle in double-mutant cell lines

**Figure 3:**
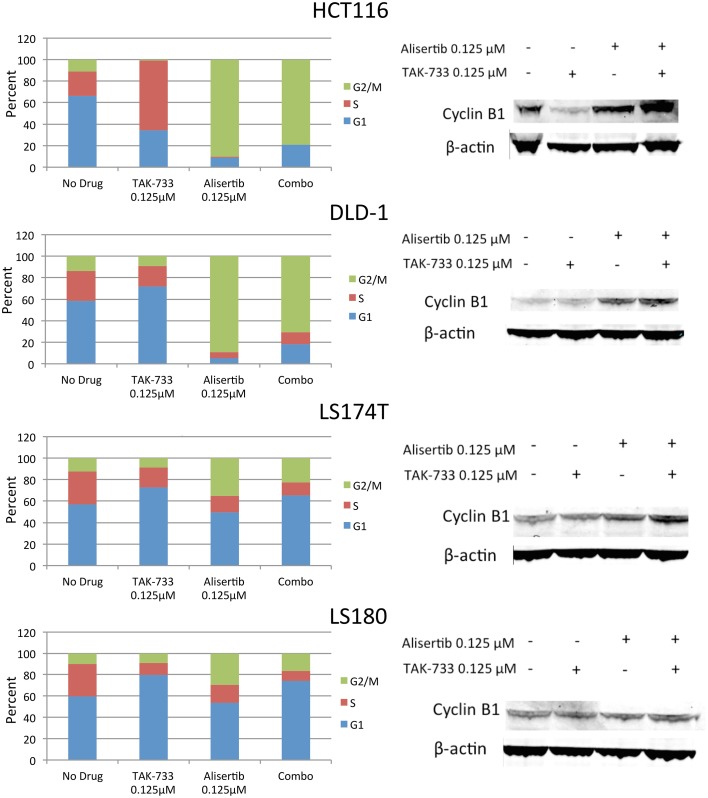
**Effect of alisertib and TAK-733 alone and in combination on the cell cycle**. **Left:** Cell cycle analysis of double-mutant CRC cell lines upon exposure to alisertib and TAK-733 as single agents and in combination for 24 h by flow cytometry, with more pronounced G2/M delay observed in HCT116 and DLD-1 cell lines. **Right:** Immunoblotting for cyclin B1 and phosphorylated cyclin B1 in double-mutant cell lines, with increase in cyclin B1 observed upon exposure to alisertib alone for 24 h, and further when combined with TAK-733 for 24 h, in 3 of 4 cell lines.

The four selected CRC cell lines were evaluated for effects on cell cycle by flow cytometry following treatment with single-agent alisertib, single-agent TAK-733, or a combination (Figure 3). Prior studies have demonstrated a delay in G2/M progression associated with Aurora A kinase inhibition of alisertib (Manfredi et al., 2011), while MEK inhibition by TAK-733 has been shown to produce cell cycle arrest in G1 (von Euw et al., 2012). Similar to other reports, we demonstrated a robust G2/M delay in HCT116 and DLD-1 following treatment with alisertib, which was largely conserved with the drug combination. However, this G2/M delay was not as significant in LS174T and LS180, indicating a differential effect on cell cycle activity in these cell lines.

To confirm these findings, additional markers of cell cycle progression were evaluated by immunoblotting (Figure 3). An increase in total cyclin B1, a mediator of mitotic entry, was observed in HCT116, DLD-1, and LS180 cells following treatment with alisertib alone and in combination with TAK-733, though no change in cyclin B1 was observed in LS174T cells. In normal human cells, levels of total cyclin B1 increase through late G2 phase, and decrease throughout mitosis (Lindqvist et al., 2009).

#### Apoptosis in colorectal cancer cell lines treated with alisertib and TAK-733

To evaluate the effects of these drugs on apoptosis, a caspase 3/7 assay was performed at 24, 48, and 72 h (Supplemental Data Image 3A). Activity of caspase 3/7 increased in DLD-1 (representative of a cell line with more robust G2/M delay per data above) with alisertib alone at 0.125 and 0.5 μM concentrations, and in combination with TAK-733 0.0625 μM at 48 h. At 72 h, caspase activity in the cells treated with all three doses of alisertib (0.03125, 0.125, and 0.5 μM) in combination with TAK-733 (0.0625, 0.125, and 0.25 μM) increased above that in cells treated with alisertib alone. No significant increase in caspase 3/7 was observed in LS174T (representing a cell line with less pronounced G2/M delay) at both 48 and 72-h time points.

Immunoblotting for cleaved PARP as an additional apoptotic marker also demonstrated distinct responses in the DLD-1 vs. LS174T cell lines when treated with alisertib alone and in combination for 24 h (Supplemental Data Image 3B). A significant increase in cleaved PARP was demonstrated in the DLD-1 cell line treated with alisertib, which further increased when this drug was combined with TAK-733. No cleaved PARP was observed in the LS174T cell line when treated with alisertib or the drug combination.

**Figure 4:**
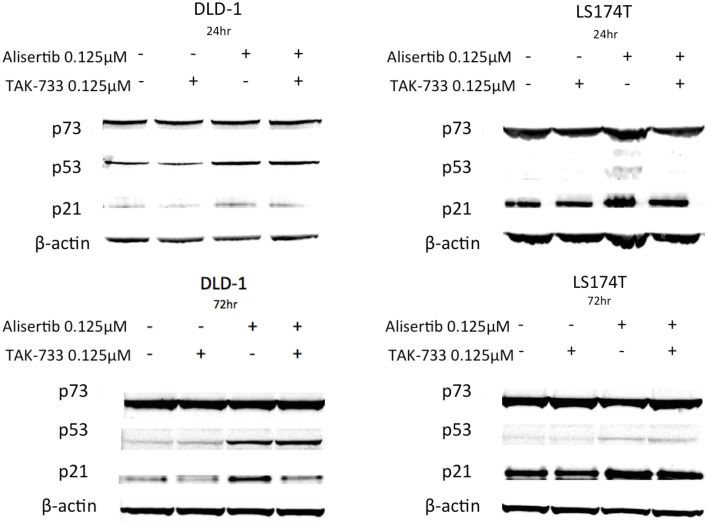
**Effect of alisertib and TAK-733 alone and in combination on p53, p73, and p21**. Evaluation was performed in p53 mutant (DLD-1) and wild type (LS174T) CRC cell lines at 24 and 72 h, with a more robust increase in p53 observed at both 24 and 72 h in the p53 mutant DLD-1 cell line. An increase in p21 is demonstrated in the DLD-1 cells exposed to alisertib alone, with no change noted in the LS174T cell line.

To further evaluate the mechanisms underlying the apoptotic activities documented above, the tumor suppressor protein p53 and its homolog p73—both generally associated with pro-apoptotic activity (Melino et al., 2002; Moll et al., 2005)—as well as p21, a Cdk inhibitor and downstream effector of p53, were evaluated by immunoblotting (Figure 4). Again using the same cell lines to compare models in which treatment with alisertib and TAK-733 produced differing apoptotic effects, p53 was noted to increase upon treatment with alisertib alone and in combination with TAK-733 in the p53 mutant cell line DLD-1 at both 24 and 72 h. However, in the p53 wild type cell line LS174T, a slight increase in p53 was observed at 72 but not at 24 h. Similarly, p21 increased slightly in the alisertib-treated DLD-1 cells at 24 h, though higher levels of p21 are present and relatively stable in both treated and untreated LS174T cells at this time point. At 72 h, p21 appears to increase in the DLD-1 cells treated with alisertib alone, while a modest increase is seen in LS174T cells treated with the drug alone and in combination. No difference was observed in levels of p73 regardless of treatment.

### *In vivo* effects of Aurora A kinase and MEK inhibitors

#### *In vivo* modeling to assess combination effects in colorectal cancer cell line xenografts

To further evaluate the efficacy of the combination of alisertib and TAK-733 *in vivo*, colorectal cancer cell line xenograft experiments were performed. Two double-mutant cell line xenografts (HCT116 and HCT15), and one *BRAF* mutant (COLO741) were treated with alisertib and TAK-733 alone and in combination. Each tumor was modeled individually, and the mean of each individual fit is presented in Supplemental Data Image 4A (Bradshaw-Pierce et al., 2013). Based on this modeling, a mathematical assessment of synergistic, additive, or antagonistic response to combination therapy with alisertib and TAK-733 could be assessed. According to the mathematical term (ψ) identified for every individual tumor for each model (see Supplemental Data Sheet 1), an additive to synergistic response to combination therapy was seen in the double-mutant HCT116 xenograft (average ψ = 1.44 ± 0.42), while a generally additive response was noted in the double-mutant xenograft HCT15 (average ψ = 1.15 ± 0.37), and less than additive effects were observed in the *BRAF* mutant COLO741 xenograft (average ψ = 0.71 ± 0.54) (Supplemental Data Image 4B).

#### Anti-tumor activity of alisertib and trametinib in patient derived colorectal xenograft models

In order to provide the most clinically relevant assessment of the combination of an Aurora A kinase and MEK inhibitor in human tumor xenograft experiments, the MEK inhibitor trametinib, which is approved for clinical use in *BRAF*-mutated, advanced melanoma (Flaherty et al., 2012), was substituted for TAK-733. Thirty colorectal cancer cell lines treated with trametinib demonstrated a pattern of anti-proliferative activity similar to that of TAK-733, indicating similar spectrum of activity. This panel included eight of the nine double-mutant cell lines evaluated by proliferation assays as described above (data not shown).

**Figure 5:**
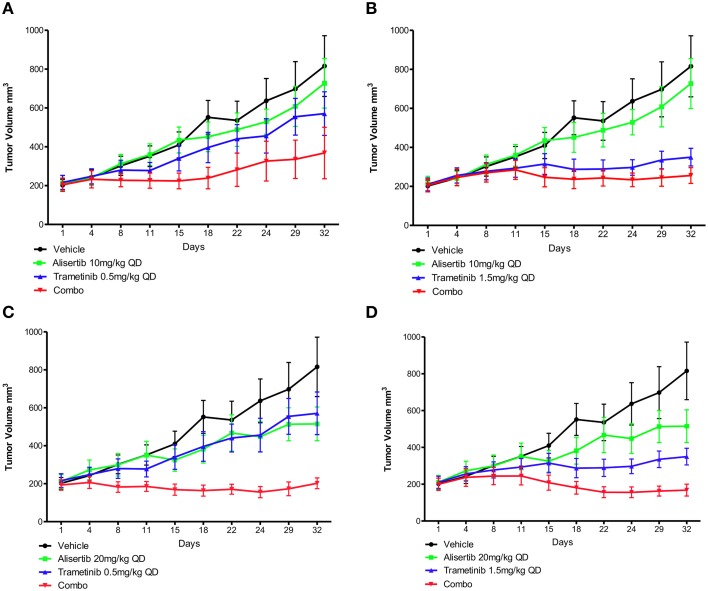
**Anti-tumor activity of alisertib and trametinib alone and in combination in CRC patient-derived tumor xenograft model CUCRC40 at various doses**. **(A)** Alisertib 10 mg/kg, trametinib 0.5 mg/kg, **(B)** alisertib 10 mg/kg, trametinib 1.5 mg/kg, **(C)** alisertib 20 mg/kg, trametinib 0.5 mg/kg, **(D)** alisertib 20 mg/kg, trametinib 1.5 mg/kg. Most notable combination effect was observed at doses of alisertib 20 mg/kg with trametinib 0.5 or 1.5 mg/kg, with a statistically significant difference between single-agent and combination treatment effect documented according to an unpaired *t*-test in these models.

Two human tumor xenograft models with known *KRAS* and *PIK3CA* mutations (CUCRC40 and CUCRC98) were treated with alisertib and trametinib as single agents and in combination at various doses: alisertib 10 and 20 mg/kg and trametinib 0.5 and 1.5 mg/kg. In the CUCRC40 model (Figures 5A–D), more pronounced tumor growth inhibition was observed with combination therapy at doses of alisertib 20 mg/kg and trametinib 0.5 mg/kg (Figure 5C). A statistically significant difference was documented between the control and combination arms (*p* < 0.001), as well as single-agent trametinib and combination arms (*p* < 0.05), at these doses according to a non-parametric Kruskal-Wallis test with Dunn's post-test. When comparing treatment arms using an unpaired *t*-test, a statistically significant difference between combination therapy and each single agent treatment was observed at these doses, as well as at doses of alisertib 20 mg/kg and trametinib 1.5 mg/kg (Figure 5D). Similar results were not observed at the lower (10 mg/kg) dose of alisertib.

This enhanced combination effect was not as pronounced in the CUCRC98 explant model (Supplemental Data Images 5A–D), where a statistically significant difference was only observed between the control and combination arms at the alisertib 20 mg/kg and trametinib 1.5 mg/kg dose (Supplemental Data Image 5D) according to a non-parametric Kruskal-Wallis test with Dunn's post-test (*p* < 0.05). This finding was confirmed by unpaired *t*-test (*p* = 0.0462).

## Discussion

Retrospective data demonstrating a lack of response of patients with *KRAS* exon 2 mutant, and now extended RAS mutant, metastatic colorectal cancer to anti-EGFR therapy has changed the focus of colorectal cancer treatment to one of personalized cancer care. Unfortunately, before these differential responses to therapy were realized, thousands of colorectal cancer patients with *KRAS* exon 2 and other clinically significant *RAS* mutations had been treated with an agent that is now known to provide them no benefit. This evolution of EGFR inhibitor therapy in the treatment of metastatic colorectal cancer has revealed the importance of identifying patient populations more likely to respond to novel biologic targeted agents in the early stages of their development, so as to avoid unnecessary exposure of patients to ineffective therapies. In line with this goal, the purpose of this study was to identify a molecular subtype of colorectal cancer that might be more likely to respond to the novel combination of a MEK and Aurora A kinase inhibitor in pre-clinical models.

The first phase of this goal was achieved in the identification of greater synergy of Aurora A kinase inhibitor alisertib and MEK inhibitor TAK-733 in CRC models with concomitant *KRAS* and *PIK3CA* mutations. Within this double-mutant subgroup, attempts were made to further define a population in which greater synergistic combination effects, rather than additive or antagonistic effects, were consistently demonstrated, as has been achieved in previous work by our group (Spreafico et al., 2013). However, despite evaluation by a variety of methods, no clear subpopulation of double-mutant colorectal cancer cell lines emerged as one associated with a more synergistic response to combination therapy.

Though reliable differences in synergistic drug effects of alisertib and TAK-733 in the double-mutant colorectal cancer cell lines were not observed, additional attempts at better defining a molecular subtype most likely to respond to therapy with this drug combination did provide interesting results. Of greatest interest are the differing effects of the drugs alone and in combination on cell cycle progression. This was demonstrated in cell cycle analysis of double-mutant cell lines by flow cytometry, where a more pronounced G2/M delay was identified in the HCT116 and DLD-1 cells treated with alisertib alone and in combination with TAK-733 as compared to the LS174T and LS180 cells.

Furthermore, a difference in levels of cyclin B1, an indicator of the outcome of cell cycle delay induced by Aurora A kinase inhibitor alisertib, was also observed in double-mutant colorectal cancer cell lines. As has been previously described, when cyclin B1 levels decline to the point that mitotic arrest can no longer be sustained, mitotic slippage (and potentially cell survival), rather than cell death, can occur if apoptotic pathways have not had sufficient time for activation (Hilton and Shapiro, 2014). However, if cyclin B1 levels can be sustained long enough, cells in which Aurora A kinase is inhibited will proceed to apoptosis. This concept is of interest to our data given the increase in cyclin B1 noted in 3 out of 4 cell lines treated with alisertib alone. Of even greater interest is the further increase in cyclin B1 levels (most clearly in the HCT116 cell line) when TAK-733 is added to alisertib as combination therapy. This finding may suggest a mechanism by which the combination of targeted agents might better facilitate apoptosis and avoid mitotic slippage reflected by a more robust increase in cyclin B1.

Though this suggestion provides one possible mechanism through which an Aurora A kinase inhibitor and MEK inhibitor may act synergistically in select colorectal cancer models, there is also data indicating that MEK inhibition induces degradation of c-Myc (Duncan et al., 2012). Though demonstrated in breast cancer models, this concept has important implications given a potential relationship between Aurora A kinase and Myc, as demonstrated in various Myc-driven malignancies including hepatocellular carcinoma and B-cell lymphomas (Den Hollander et al., 2010; Lu et al., 2014). In both tumor types, Myc has been found to induce Aurora A kinase, and thus it may be hypothesized that the more synergistic combination effects demonstrated in select CRC models could be due to MEK inhibitor mediated degradation of c-Myc leading to more robust effects on Aurora A.

The above-described *in vitro* data were further elucidated *in vivo*, using cell line and patient-derived xenograft models. Results from synergy modeling of cell line xenografts demonstrating a trend to a more synergistic response to combination therapy with alisertib and TAK-733 in one of the double-mutant models (HCT116), as compared to an additive response in the other (HCT15), is in keeping with the concept of a subpopulation within the double-mutant subgroup in which more synergistic combination effects occur, though the effect size was small. In the CUCRC40 double-mutant patient-derived tumor xenograft model, the combination of alisertib and trametinib with greatest anti-tumor activity was achieved with a higher dose of alisertib (20 mg/kg), suggesting cell cycle inhibition by the Aurora A kinase inhibitor is an important driver of tumor growth inhibition. However, the same was not observed in treatment of the CUCRC98 xenograft model, where a significant increase in tumor growth inhibition in response to combined therapy was not observed at any dose. This pattern documented across *in vivo* models indicates that, similar to patients (Amado et al., 2008; Karapetis et al., 2008; Kopetz et al., 2010), the double-mutant genotype does not identify a consistently responsive subset of CRC. It is important to note that the clinically approved MEK inhibitor trametinib was substituted for TAK-733 in these *in vivo* studies, though *in vitro* data indicating similar anti-proliferative activity across a range of CRC cell lines suggests this change did not have a drastic impact.

An additional finding of interest in our work is related to the p53 protein. The interaction between p53 and Aurora kinases are complex, and it is currently believed that Aurora A phosphorylates and thus down regulates p53 and its associated apoptotic activity (Katayama et al., 2004). In turn, inhibition of Aurora A with drugs such as alisertib leads to activation of p53 and its downstream effectors. Interestingly, loss of p53 is also thought to increase sensitivity to both Aurora A and Aurora B kinase inhibition (Marxer et al., 2014). Our assessment of p53 by immunoblotting in double-mutant colorectal cancer cell lines demonstrated an increase in p53 upon treatment with alisertib alone and in combination with TAK-733 at both 24 and 72 h in a p53 mutant model, DLD-1. Interestingly, in the p53 wild type cell line LS174T, p53 was not up-regulated after 24 h, though it was when treated with alisertib as a single agent and in combination with TAK-733 at the 72 h timepoint. These findings are consistent with prior data showing that unlike wild type p53, mutant p53 is not down-regulated by Aurora A kinase (Katayama et al., 2004). This explains the presence of p53 protein at both early and late time points in the p53 mutant cell line DLD-1 at baseline, as well as the more significant increase of the protein upon treatment with alisertib alone or in combination with TAK-733. Wild type p53 is typically undetectable by immunoblot due to its down-regulation through MDM-2. However, we observed an increased level of p53 protein in response to treatment with alisertib in the p53 WT cell line LS174T, indicating a DNA damage response as a result of prolonged cell cycle delay.

As the downstream transcriptional target of p53, p21 does slightly increase, though this appears to occur at a lesser degree in the p53 mutant DLD-1 cell line when alisertib is combined with TAK-733. This is of particular interest given the increased understanding of the duality of the p21 protein, which is currently believed to contribute to both cell cycle arrest and anti-apoptotic processes (Gartel and Tyner, 2002). The findings of a less robust increase in p21 with an equivalent increase in p53 in cells treated with combination therapy vs. alisertib alone may indicate a mechanism by which the MEK inhibitor facilitates the pro-apoptotic effects of p53 induction through Aurora A kinase inhibition by decreasing the anti-apoptotic effects of p21. This concept is further supported by the increased apoptotic activity demonstrated in the p53 mutant cell line DLD-1 when treated with the combination of alisertib and TAK-733 as compared to the p53 wild type cell line LS174T.

Also of interest is the lack of increase in p21 noted in the p53 wild type LS174T cell line upon exposure to alisertib alone and in combination, as compared to the p53 mutant DLD-1 cell line. When this finding is considered in the context of a recently identified transcription factor (TCF3/E2A) that increases expression of p21 while decreasing that of p53 target PUMA, a mediator of p53-induced apoptosis, (Andrysik et al., 2013) it seems possible that there may be an imbalance present in some molecular subtypes of CRC toward p21 activation and cell cycle arrest vs. PUMA activation and apoptosis. It is thus possible that differing levels of p21 in these cell lines indicate a trend toward one of these two subtypes. This concept is in line with findings in our work that demonstrate a more pronounced G2/M delay in the DLD-1 cells upon exposure to alisertib alone and in combination with TAK-733 that mirrors the increase in p21, while in LS174T cells where less pronounced G2/M delay was documented, no change in p21 was identified. This data may indicate that DLD-1 and LS174T cell lines do in fact represent distinct molecular subtypes of CRC.

Our data represents the first evaluation of the combination of an Aurora A kinase and MEK inhibitor in colorectal cancer models. Unfortunately, as has been a common theme in clinical studies evaluating targeted agents for the treatment of metastatic colorectal cancer, we were unable to clearly identify a subset of CRC that is more (or more consistently) responsive to the combination of a MEK and Aurora A kinase inhibitor. Though greater elucidation of specific biomarkers predictive of response to such combination therapy was not achieved in our work, our efforts do suggest that degree of G2/M cell cycle delay, p53 mutation status, and possibly p21 levels, may affect outcomes of treatment with the drug combination on the cellular level, and provide areas of further investigation for pre-clinical and clinical studies alike. Moving forward, further development of predictive biomarkers will be key in determining the clinical applicability of this drug combination.

## Author contributions

Conceived and designed the experiments: TP, JT, EBP, JE, SE. Performed the experiments: SD, KR, TP, EP, PK, SB, SH, HS, AS, JA. Analyzed the data: SD, TP, JT, EP, AT, SE. Contributed reagents/materials/analysis tools: TP, JA, WM, AT, SE. Wrote the paper: SD, TP, EP, SE.

### Conflict of interest statement

This work was supported by Takeda Pharmaceuticals and University of Colorado Cancer Center Grant P30 CA046934. The funders had a minor role in study design and decision to publish. The funders had no role in data collection and analysis, or preparation of the manuscript. Erica L. Bradshaw-Pierce and Jeffrey A. Ecsedy are current employees of Takeda. The authors declare that the research was conducted in the absence of any commercial or financial relationships that could be construed as a potential conflict of interest.
