# Supplementary material for: Combined inhibition of MEK and Aurora A kinase in KRAS/PIK3CA double-mutant colorectal cancer models
Source: Front Pharmacol. 2015 Jun 16;6:120. doi: 10.3389/fphar.2015.00120 (PMC4468631; doi:10.3389/fphar.2015.00120)
Supplement: Supplementary file 1 [file DataSheet1.PDF]

Supplementary Methods

Combination Modeling

The combination of alisertib and TAK-733 therapy was determined by mathematical modeling of the tumor growth profiles of individual tumors similar to a previously published method [24]. All HCT116, HCT15 and COLO741 xenograft control (vehicle treated) tumors were individually fit to an exponential growth rate equation:

$$\frac{dT}{dt} = k_g * T; \;\; T(0) = T_0$$

where T is the tumor volume (mm^3) with respect to time, k_g is the exponential tumor growth parameter, and T_0 is the estimated initial tumor volume (at time = 0). The individual parameters (k_g and T_0) were averaged and the final parameter values are presented in **Table 1**. The average growth terms for each tumor type were then fixed at the average parameter value and used to determine the single agent effects (k_x) of each compound by fitting the following equation:

$$\frac{dT}{dt} = k_g T - k_x c_x T, \quad T(0) = T_0$$

where c_x is the concentration of drug x with respect to time. Individual tumors were modeled and the average value of k_x determined (**Table 1**) for each single agent treatment. This value was used to fit the following equation to the combination tumor growth data to determine the degree of interaction between the two drugs.

$$\frac{dT}{dt} = k_g T - [\, k_1 c_1 T + \; k_2 c_2 T\square], \quad T(0) = T_0$$

The interaction term ψ was determined for each individual tumor for each cell line xenograft model, as shown in Supplemental Figure 5B. We determined that ψ values greater than 1.3 are

synergistic, between 1.3 and 0.7 are additive, between 0.7 and 0 are less than additive, and negative values for ψ are antagonistic. These ranges were selected based on the model fits. The CV% for the model fits ranged from 9 to 91 with an average CV% of 30% (SD=23%). Therefore we assume an average variance of 30% making the additive range from 1.3 to 0.7 (1 + 30% and 1- 30%).

Table 1. Combination Modeling Parameter Values

		k_g [h-1]	T_0 [mm3]	k_1	k_2
HCT15	Control	0.00311 (0.00078)	94.7 (59.3)		
	Alisertib			-8.41E-7 (5.41E-6)	
	TAK7-33				1.79E-5 (1.06E-5)
HCT116	Control	0.00415 (0.00197)	82.9 (49.9)		
	Alisertib			1.98E-6 (9.39E-6)	
	TAK7-33				2.04E-5(1.17E-5)
COLO741	Control	0.00231 (0.00088)	64.2 (29.4)		
	Alisertib			2.65E-06 (4.38e-6)	
	TAK7-33				5.69E-06 (4.09E-06)

Mean data (SD)

Pharmacokinetic (PK) models were generated for alisertib and TAK-733. Data used to fit the alisertib and TAK-733 pharmacokinetics was obtained in the study presented here. Alisertib PK was described by a two compartment PK model with first-order absorption and TAK-733 PK in this study was described by a one-compartment model with first-order absorption. **Tables 2-5** contain the parameter values for the model fits and **Supplementary Methods Figure 1** shows the compartmental model structures used.

Table 2. Alisertib Single Agent PK Model Fits

	Parameter	Value	StDev	CV
HCT 15	V [mL/kg]	263.2	42.32	16.1
	kel [h-1]	2.54	0.30	11.9
	ka [h-1]	7.57	3.18	42.0
	k(2,1) [h-1]	0.33	0.06	16.7
	k(1,2) [h-1]	3.52	0.94	26.8
HCT 116	V [mL/kg]	278.2	40.15	14.4
	kel [h-1]	2.55	0.29	11.2
	ka [h-1]	8.38	3.17	37.9
	k(2,1) [h-1]	0.29	0.05	15.9
	k(1,2) [h-1]	4.39	1.07	24.4
COLO741	V [mL/kg]	265.3	42.35	16.0
	kel [h-1]	2.33	0.30	12.8
	ka [h-1]	5.32	2.12	39.7
	k(2,1) [h-1]	0.21	0.05	22.9
	k(1,2) [h-1]	4.76	1.28	27.0

Table 3. Alisertib Combination PK Model Fits

	Parameter	Value	StDev	CV
HCT 15	V [mL/kg]	274.4	42.04	15.3
	kel [h-1]	2.48	0.30	11.9
	ka [h-1]	6.79	2.68	39.5
	k(2,1) [h-1]	0.27	0.05	18.4
	k(1,2) [h-1]	4.29	1.13	26.4
HCT 116	V [mL/kg]	292.7	45.34	15.5
	kel [h-1]	2.68	0.31	11.6
	ka [h-1]	8.79	3.54	40.3
	k(2,1) [h-1]	0.29	0.06	19.8
	k(1,2) [h-1]	4.32	1.17	27.1
COLO741	V [mL/kg]	268.0	41.91	15.6
	kel [h-1]	2.45	0.30	12.2
	ka [h-1]	8.03	3.40	42.4
	k(2,1) [h-1]	0.27	0.05	19.0
	k(1,2) [h-1]	4.70	1.20	25.6

Table 4. TAK-733 Single Agent PK Model Fits

	Parameter	Value	StDev	CV
HCT15	V [mL/kg]	1835	524.4	28.6
	kel [h-1]	0.72	0.21	29.5
	ka [h-1]	0.103	0.035	34.0
HCT116	V [mL/kg]	1631	504.6	30.9
	kel [h-1]	0.59	0.20	33.7
	ka [h-1]	0.104	0.035	34.1
COLO741	V [mL/kg]	1388	419.2	30.2
	kel [h-1]	0.62	0.20	31.9
	ka [h-1]	0.109	0.035	31.8

Table 5. TAK-733 Combination PK Model Fits

	Parameter	Value	StDev	CV
HCT15	V [mL/kg]	2196	537.3	24.5
	kel [h-1]	0.69	0.18	25.6
	ka [h-1]	0.094	0.028	29.5
HCT116	V [mL/kg]	1599	434.4	27.2
	kel [h-1]	0.68	0.19	27.3
	ka [h-1]	0.105	0.030	28.7
COLO741	V [mL/kg]	1736	527.2	30.4
	kel [h-1]	0.54	0.18	33.1
	ka [h-1]	0.108	0.034	31.5

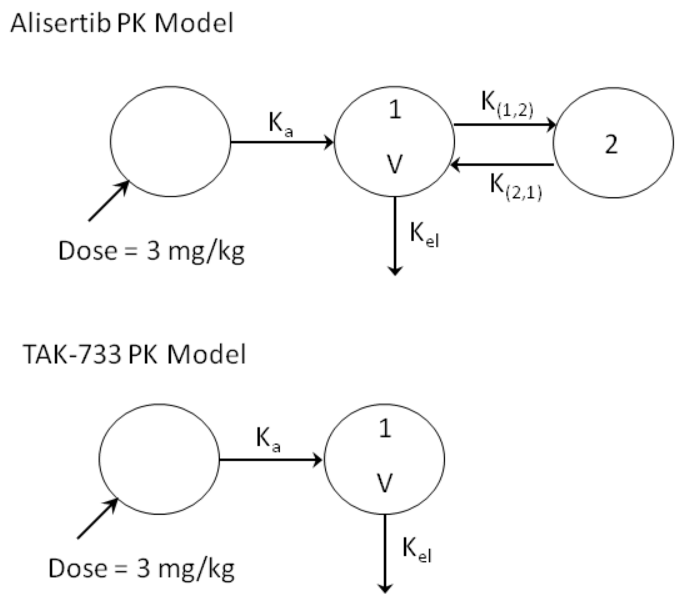

Supplementary Methods Figure 1. Compartmental model structures for alisertib and TAK-733. These PK models were used to simulate alisertib and/or TAK-733 plasma concentrations over time for combination modeling.
