# Supplementary figures and images for: Combined inhibition of MEK and Aurora A kinase in KRAS/PIK3CA double-mutant colorectal cancer models
Source: Front Pharmacol. 2015 Jun 16;6:120. doi: 10.3389/fphar.2015.00120 (PMC4468631; doi:10.3389/fphar.2015.00120)

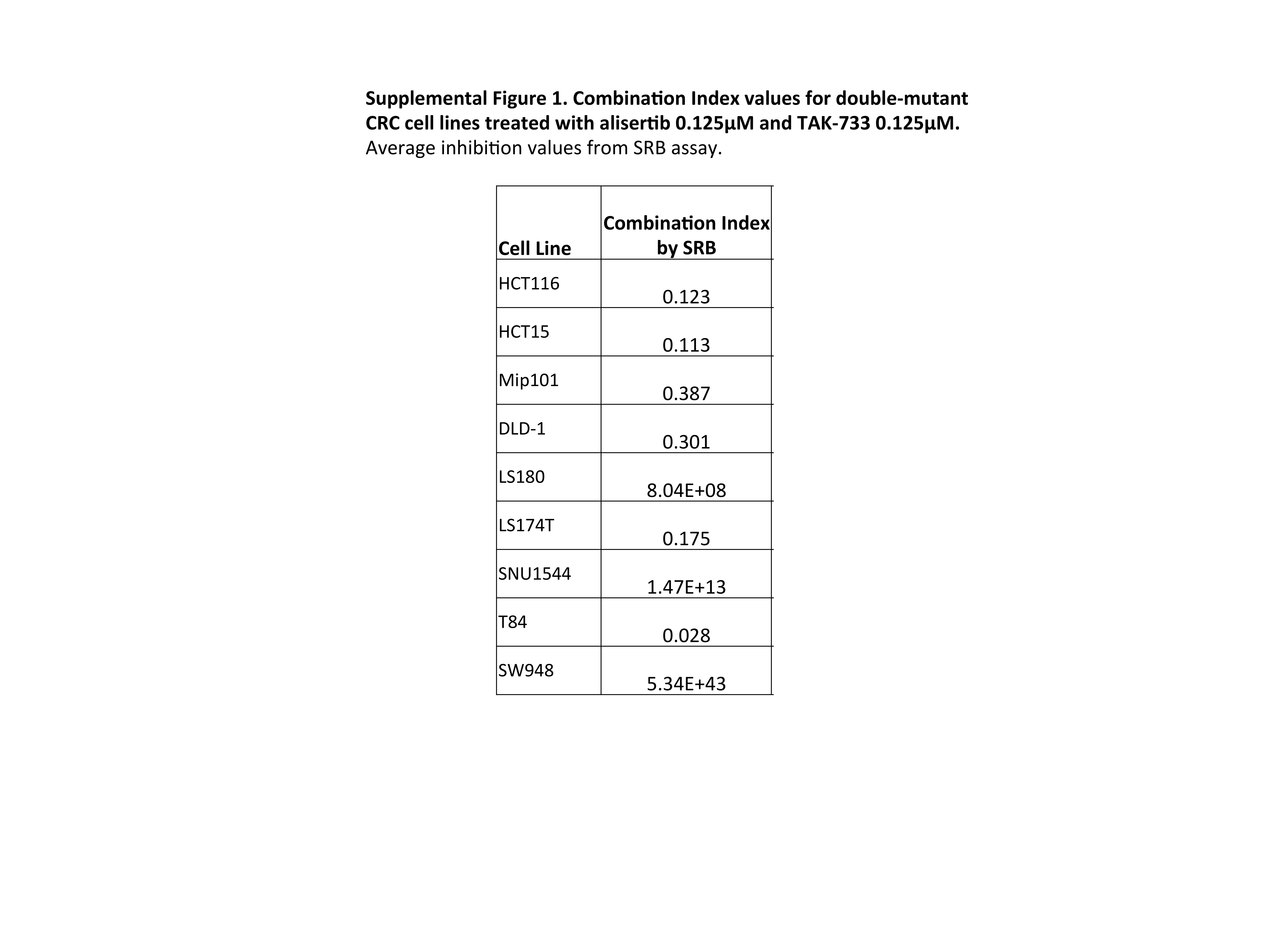

Supplement: Supplementary file 2 [file Image1.TIF]

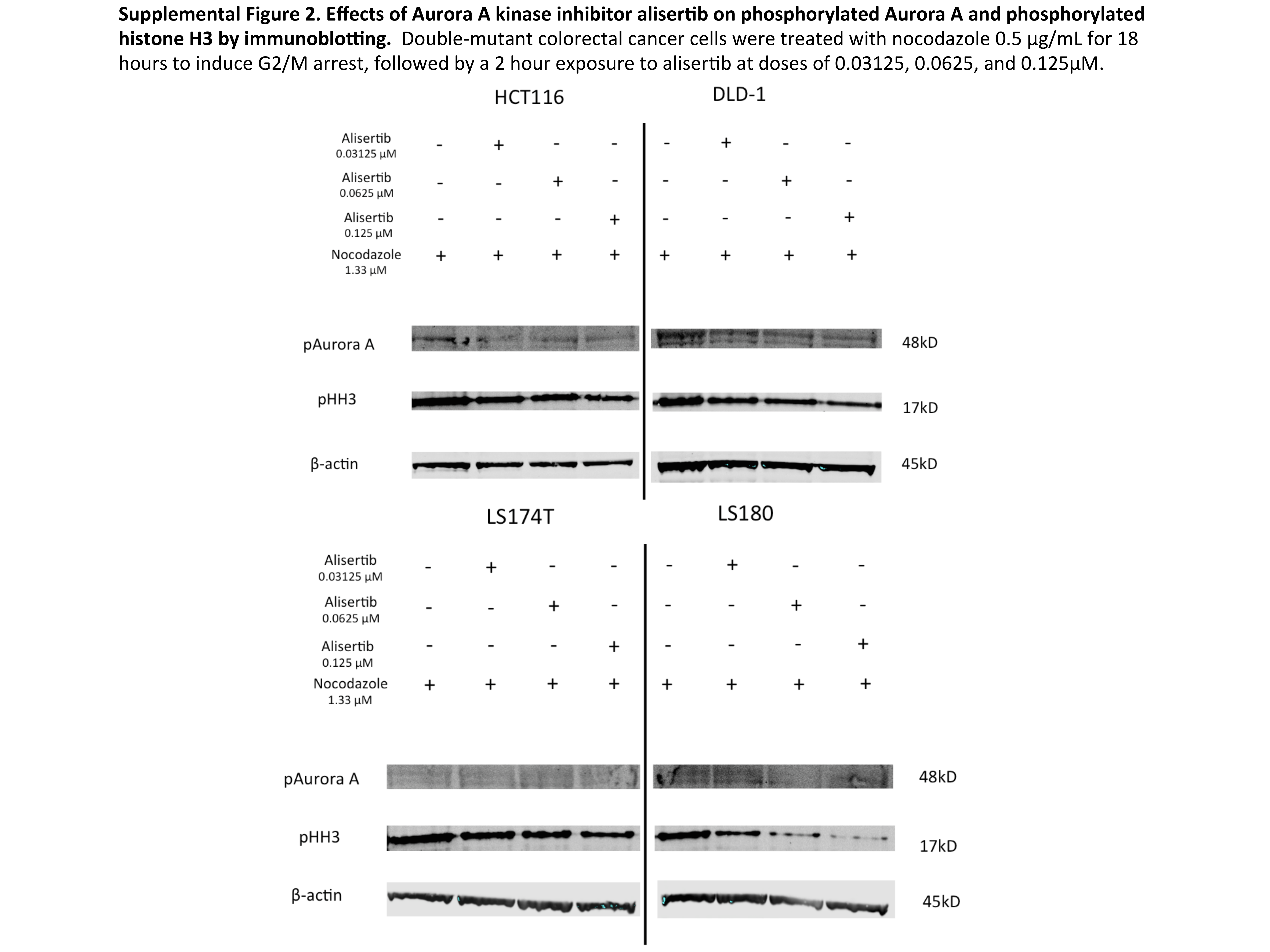

Supplement: Supplementary file 3 [file Image2.TIF]

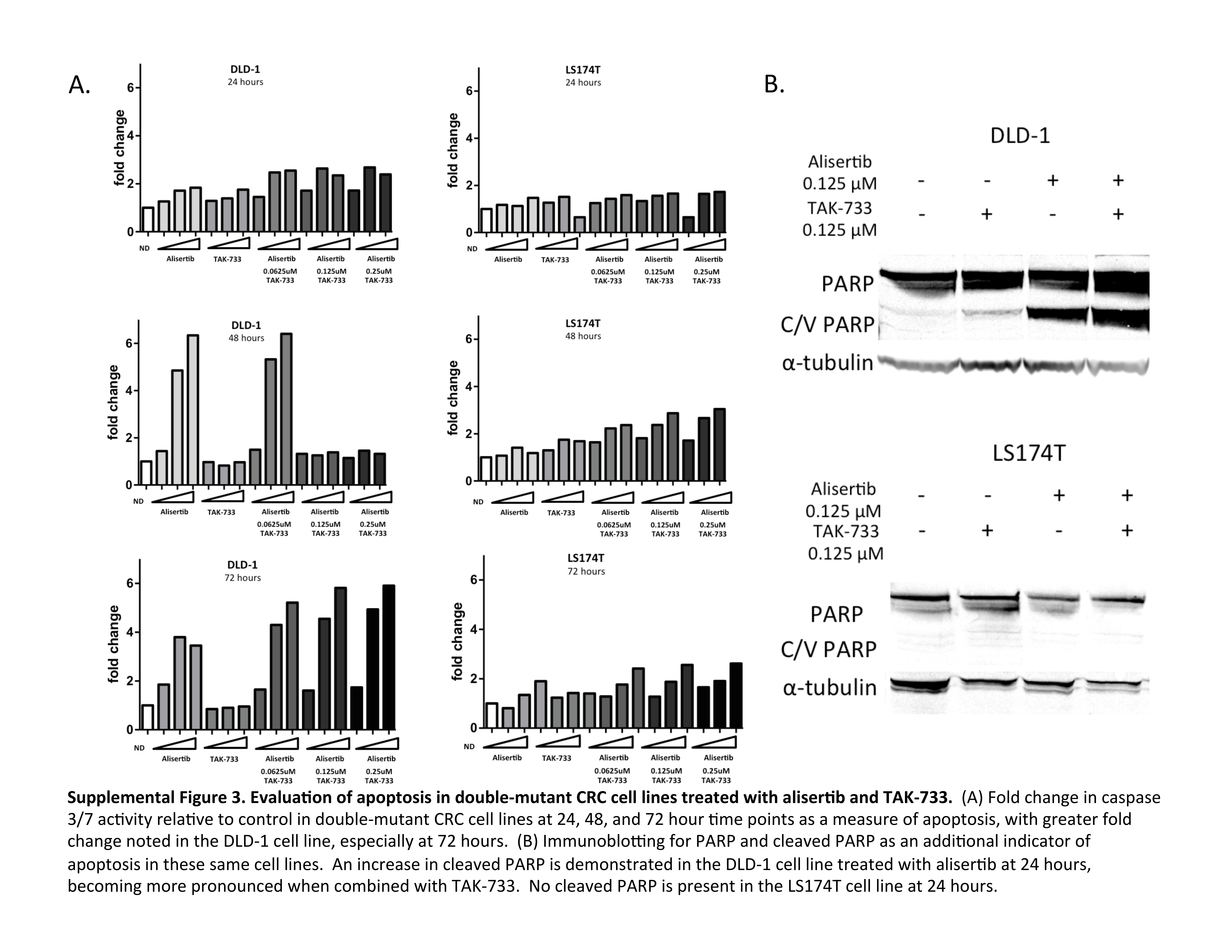

Supplement: Supplementary file 4 [file Image3.TIF]

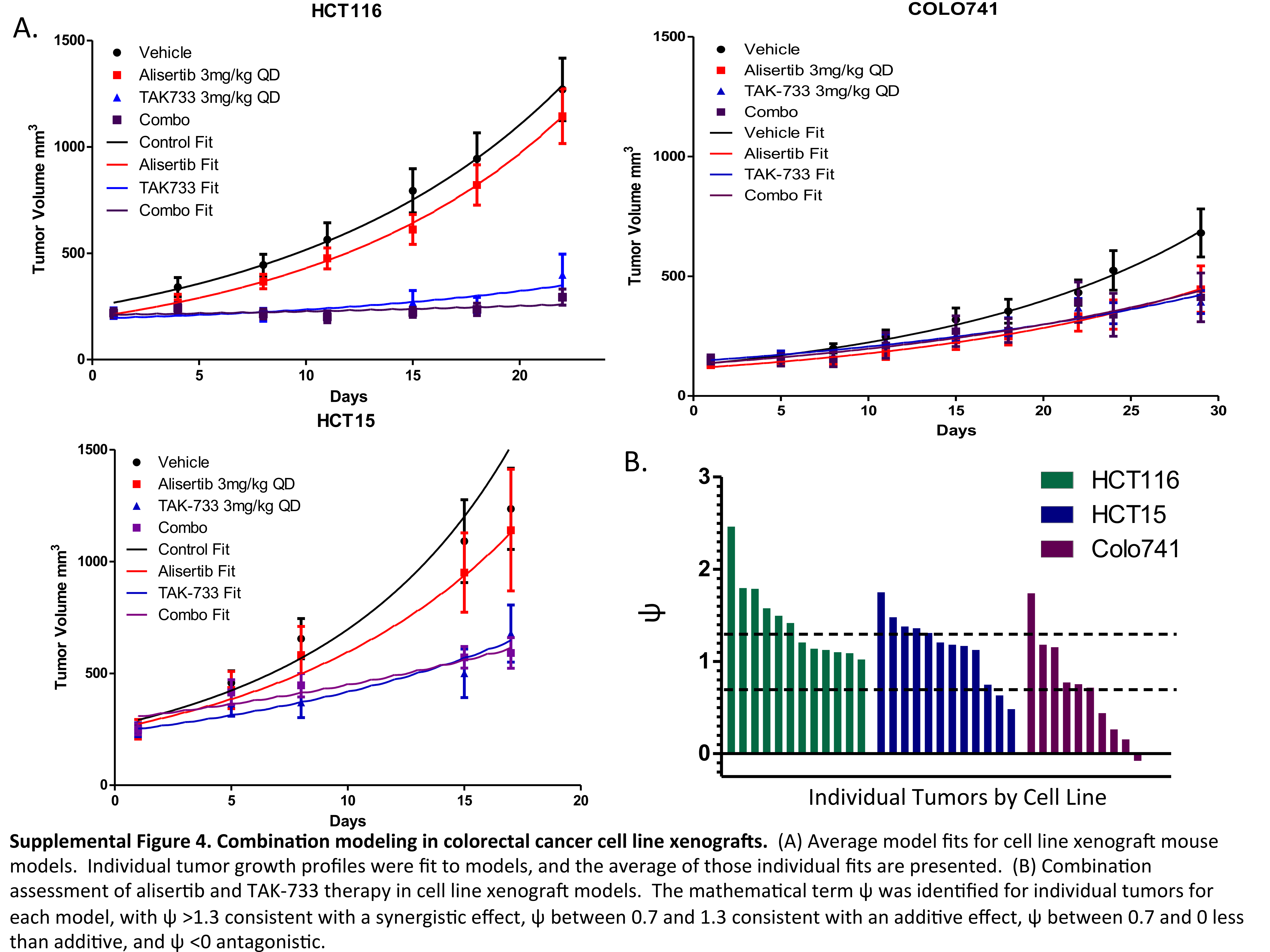

Supplement: Supplementary file 5 [file Image4.TIF]

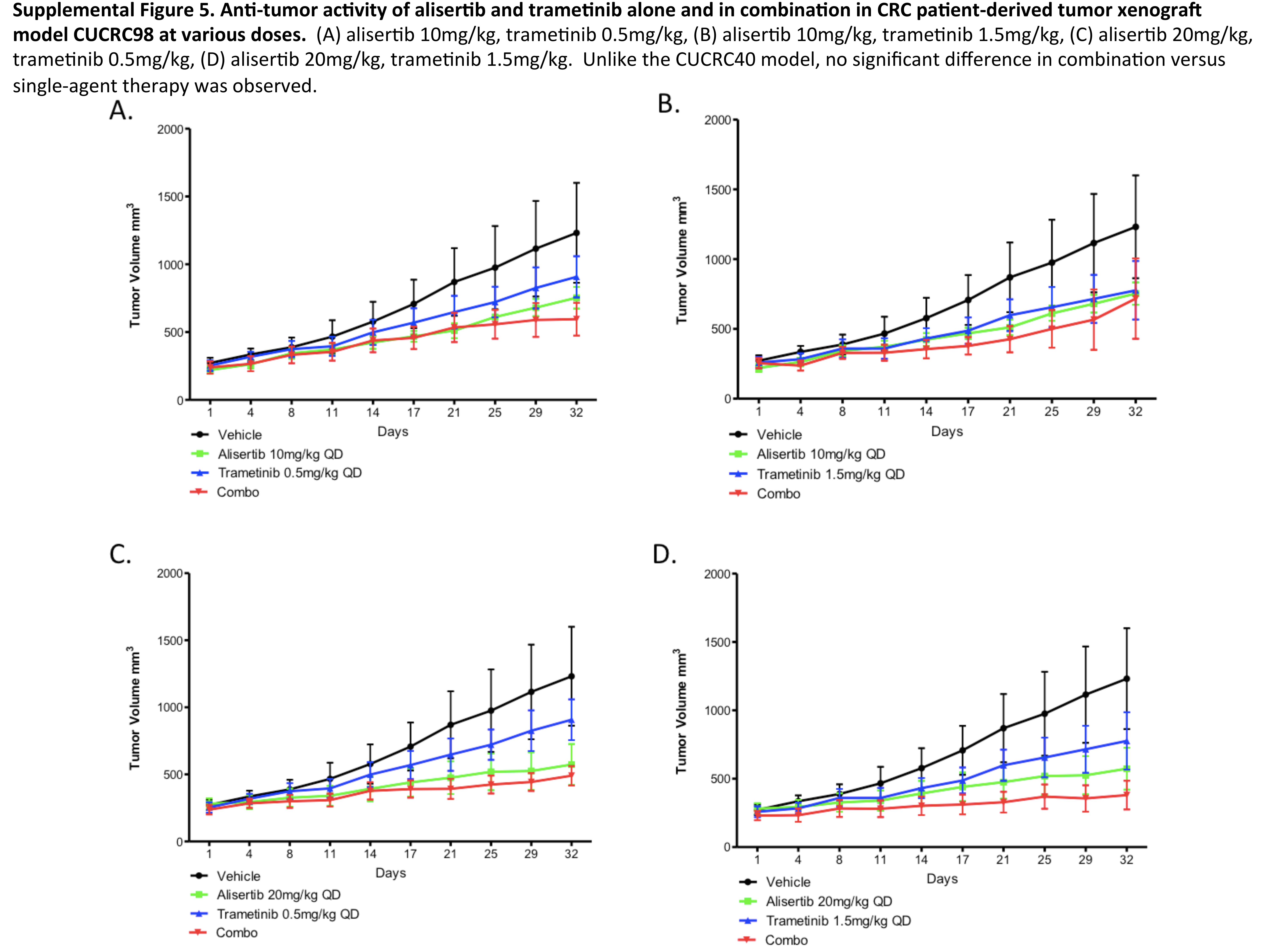

Supplement: Supplementary file 6 [file Image5.TIF]
